# Relationship between effects on time-to-disease progression and overall survival in studies of metastatic breast cancer

**DOI:** 10.1038/sj.bjc.6604759

**Published:** 2008-10-28

**Authors:** B Sherrill, M Amonkar, Y Wu, C Hirst, S Stein, M Walker, J Cuzick

**Affiliations:** 1 RTI Health Solutions, Biometrics, Research Triangle Park, NC, USA; 2 GlaxoSmithKline, Global Health Outcomes, Collegeville, PA, USA; 3 RTI Health Solutions, Epidemiology Group, Barcelona, Spain; 4 Department of Oncology, GlaxoSmithKline, Collegeville, PA, USA; 5 GlaxoSmithKline, Global Health Outcomes, London, UK; 6 Department of Mathematics and Statistics, Cancer Research UK Centre for Epidemiology, Wolfson Institute of Preventive Medicine, Queen Mary School of Medicine and Dentistry, London, UK

**Keywords:** metastatic breast cancer, survival, disease progression

## Abstract

The relationship between overall survival (OS) and disease progression end points has been demonstrated in colorectal, colon, and non-small cell lung cancers. We assessed the association between OS and time-to-progression (TTP) or progression-free survival (PFS) in metastatic breast cancer (MBC) studies. A literature search retrieved all randomised controlled trials since 1994 in patients with MBC in which OS and either TTP or PFS were reported. Summary data on trial and patient characteristics were abstracted. Study effect sizes were derived as the ratio of median progression (or survival) times, which approximates the hazard ratio. Effects were centred at zero for regression analyses weighted by sample size. Numerous treatments were represented in 67 studies (17 081 patients). Modeling showed a positive association between outcomes for progression and survival (*R*^2^=0.30) with a slope of 0.32 (*P*<0.001) and a non-significant intercept. Thus, a treatment effect on TTP/PFS translated into a concordant effect on OS, but with attenuated effect size. Similar results were found in models of subsets and sensitivity analyses. These results demonstrate that treatment effects on progression end points in MBC trials are expected to result in treatment differences on OS that are smaller yet consistently in the same direction.

Most studies of anticancer agents are designed and powered to show differences between groups on disease progression end points such as response rates, time-to-progression, time-to-recurrence or disease-free survival. Although increased survival time is clearly an implicit goal, it is difficult to establish in randomised studies when standard clinical practise is to change treatments at the onset of new progressive events. Especially in the metastatic setting, where expected survival time may be very short, the use of drug combinations or sequential use of multiple drug classes is commonplace. Increasingly, patients in clinical trials are offered alternate treatment upon progression. Consequently, few studies actually demonstrate an unequivocal survival benefit using intent-to-treat analysis approaches.

In several tumour types, efforts have been made to justify the use of alternate, more easily established end points by showing how well they are correlated with survival. Several meta-analyses have been published (Table 1) that show a link between time-to-progression (TTP) or progression-free survival (PFS) with overall survival (OS) in metastatic colorectal or non-small cell lung cancer (Johnson *et al*, 2006) and colon cancer in the adjuvant setting (Sargent *et al*, 2005). For breast cancer, a moderately strong correlation between 2-year disease-free survival and 5-year overall survival was observed in the adjuvant setting (Ng *et al*, 2008). A meta-analysis of metastatic breast cancer studies found a positive relationship between objective response and overall survival on an individual patient basis, but the relationship was less clear when treatment regimens were compared (Bruzzi *et al*, 2005). The authors questioned whether the relationship was strong enough to support objective response rate as a valid surrogate of survival when comparing treatments. Articles by two groups (Hackshaw *et al*, 2005; Burzykowski *et al*, 2008) examined the association with survival of several potential surrogate end points, specifically in studies of anthracyclines for advanced or metastatic breast cancer.

We performed a broad-based structured review and meta-analysis of randomised controlled trials in metastatic breast cancer. The objective of this research was to document and model the relationship between time-to-disease progression and OS in metastatic breast cancer studies across the literature.

## Methods

### Study selection

Inclusion criteria for studies were determined prior to starting the literature search. Published articles of randomised controlled trials (RCTs) of patients with metastatic breast cancer were considered for inclusion if they were published in English after 1994 and reported both TTP (or PFS) and OS. Only studies in which the entire population had metastatic breast cancer (MBC) were included; studies that included patients with locally advanced breast cancer were not included to avoid potential confounding. PubMed searches were conducted using the terms ‘breast cancer,’ ‘metastatic,’ and ‘survival’ combined with recognised search terms to identify randomised controlled trials. In addition to the electronic literature search, we reviewed bibliographic reference lists of included studies and key review articles to identify other studies. Titles were initially reviewed for relevance. For those potentially meeting the inclusion criteria, the abstract was then reviewed. Finally, for those abstracts that appeared eligible for inclusion, full-text articles were retrieved and further examined.

### Data abstraction

Data were abstracted from studies where estimates were provided for both progression and survival outcomes. In addition to the summary data, we abstracted definitions of the primary outcomes in each trial. Distinctions between definitions for TTP and PFS were not consistent. Many trials included all deaths as events for the TTP outcome, which is more typically done for the PFS outcome. For this reason and considering the similarity in summary data, we combined results for the outcomes of TTP or PFS.

The methodological quality of each trial was assessed by confirming randomisation and allocation concealment, identifying whether the trial was blinded, and whether intention-to-treat analyses were available. After review of abstracted data, items were grouped or redefined to combine and use data from the most studies possible. Ratings for disease severity using Eastern Cooperative Oncology Group or Karnofsky scales were converted to the World Health Organization (WHO) scale. Where possible, studies were characterised as to whether patients were identified as ER+ and/or PR+, and also HER2+ (ER/PR=oestrogen receptor/progesterone receptor; HER2=human epidermal growth factor receptor-2). Treatments were characterised as first-line or subsequent (if any prior treatments for MBC) and grouped by drug class. For simplicity, only two treatment groups were used from each study. For studies that included three treatment groups, the group with the highest efficacy compared with control or the highest group from dose-ranging studies was included. Treatment groups were reviewed by two clinicians and identified, to the best extent possible, as an experimental treatment or a more standard treatment.

All studies provided at least the median overall survival and median TTP/PFS per study arm. We abstracted effect sizes and variances, where available. Hazard ratios and s.e. were not consistently available, and variances could not be calculated for all studies. For this reason, we show regression models weighted by sample size.

### Data analysis

Although few studies reported hazard ratios (HR), we derived it as the ratio of median times to event for the two treatment groups, under the assumption that survival follows an exponential distribution (Hackshaw *et al*, 2005). HRs were computed for experimental treatment *vs* standard treatment. The estimates for HR_TTP_ and for HR_OS_ were each centred at unity, so that the treatment effects for each outcome were defined as:

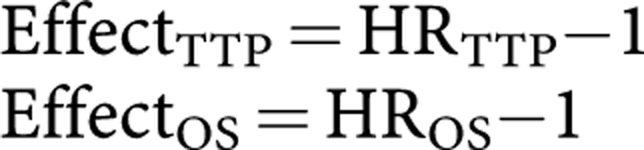


Using traditional meta-regression techniques, each study was analysed as a unit. Modeling by study provides a test for the association between each effect size for progression and effect size for survival. In other words, the model tests whether a study that shows differentiation between groups on the progression outcome is expected to show differentiation between groups on survival.

The basic model structure for the meta-regression is:

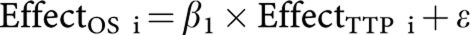
 where each study (*i*) contributed one observation to the meta-regression, weighted by sample size. Note that the intercept is forced to equal zero to reflect the plausible assumption that treatments with no effect on TTP will have no effect on survival. Preliminary modeling justified the choice of no intercept. For computing prediction intervals associated with different values of Effect_TTP_, an intercept term was included so that perfect prediction at the no effect point was not assumed. Average survival time was considered as a covariate, but it was not significant, did not affect inferences, and was removed for ease of presentation. Exclusion of outliers did not affect inferences, so all studies were retained in the analyses. Subgroups of clinical interest were modeled separately for ease of interpretation. All analyses were performed using SAS version 9.1.

## Results

**Figure 1:**
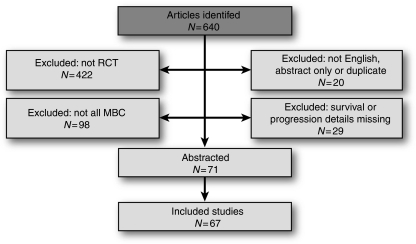
Flow diagram of literature search.

The electronic literature search was conducted using PubMed on 11 October 2007. Data flow is shown in Figure 1 and study data is presented in Appendix 1. Two articles represented data from the same clinical trial (Eiermann, 2001; Slamon *et al*, 2001), and three of the 71 abstracted articles were subsequently excluded because they used high-dose chemotherapy, which has limited application to current clinical practise (Lotz *et al*, 2005; Schmid *et al*, 2005; Kröger *et al*, 2006). The resulting 67 clinical trials represent 17 081 patients. All trials were randomised. One trial was placebo-controlled and two allocated the control group to observation or usual care. Blinding was identified for only two trials. In 37 trials, analyses were identified as intent-to-treat; other trials did not specifically state intent-to-treat approach.

Most trials required patients at entry to be rated as WHO Grade 2 or below for performance status; 11 studies allowed up to Grade 3 performance status. Median age was 55 years (range: 50–75 years). Hormone status for patients was presented for 46 studies, ranging from 14 to 84% oestrogen and/or progesterone receptor (ER/PR) positive. Only six studies provided HER2 (human epidermal growth factor receptor-2) status, four of which included only HER2+ patients. Most studies were of first-line treatment for MBC (46 studies); some of these studies allowed prior adjuvant treatment, but not prior chemotherapy for MBC. A range of drug classes were represented in 28 studies of monotherapies and 39 studies of combination regimens as shown in Tables 2 and 3.

The unweighted median TTP/PFS averaged across all treatment groups was 7 months (range, 2–15 months), and the average survival time was 20 months (range, 8–38 months). Correlation was 0.38 between time-to-progression and survival in the separate treatment groups (that is, ignoring trial comparisons).

Of 31 studies that provided tests of treatment differences for both TTP/PFS and OS, there was a moderate agreement between outcomes (Kappa test for agreement=0.47, *P*<0.05). About half of these studies (48%) had a significant effect on progression, but only 23% had a significant effect on survival. None of the studies reported a significant effect on survival if there was not a significant effect on progression.

**Figure 2:**
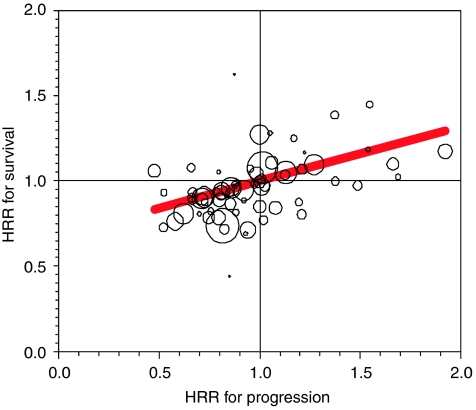
Plot of HR for survival *vs* HR for progression by study size regression line: Effect_OS_=0.32 × Effect_TTP_ where Effect is the HR centred at unity. Bubbles show relative sample sizes from each study.

Figure 2 shows HR_OS_ *vs* HR_TTP_ for all studies with the radius of the bubbles representing relative study sample sizes. The range of HR_TTP_ for comparing experimental to standard treatments was 0.5–1.9; HR_OS_ ranged from 0.4 to 1.6. Minimal effects on both progression and survival were seen in 13% of studies; that is, the hazard ratio for both end points was close to one (0.9 <HR <1.1). In 39% of studies, treatment effects on both progression and survival were greater than 10% and in the same direction. Other within trial treatment comparisons gave mixed results on the two end points. In many studies, the hazard ratios showed a treatment effect on progression with minimal effect on survival (30%); fewer studies resulted in hazard ratios representing a minimal effect on progression with a more pronounced effect on survival (13%). In the very few studies with discordant results on treatment effect (that is, HRR_TTP_ and HRR_OS_ in opposite directions, shown in upper left and lower right quadrants of Figure 2), no pattern was apparent in terms of treatment or patient types. The unweighted Pearson correlation between HR_TTP_ and HR_OS_ across trials was 0.46.

The regression line shown in Figure 2 corresponds to the primary model results shown in Table 4. If the slope coefficient in this model equals zero (*β*=0), then the treatment effect on progression is not expected to affect treatment difference in survival; if *β*=1, treatment effects on survival are expected to mirror treatment effects on progression end points. For 0< *β* <1, we would expect that a treatment effect on progression is concordant but not as large for the OS outcome. In this meta-analysis, we found *β*_1_=0.32 (95% CI: 0.20, 0.43) supporting the last interpretation (*R*^2^=0.30). Results did not suggest curvature in the relationship (quadratic term, *P*=0.16). Adding an intercept term had only a minor effect on the slope parameter. The model predicts that a treatment effect on HR for progression equal to 0.70 would translate into a hazard ratio for survival of 0.88 (Table 5). Note that the confidence intervals shown in Table 5 are based on modelling the meta-analysis data and are not intended for trial planning purposes; the width of the interval will be dependent on the sample size of future trials.

In studies of hormonal agents, the regression had a steeper slope with a wide CI encompassing one (*β*_1_=0.58; 95% CI: 0.001, 1.17; Table 4). Results in subsets of anthracycline studies, first-line treatments and later treatments were similar to the overall analysis. In four studies in which all patients were identified as HER2+, the slope was 0.50 but the CI did not encompass one. In this group of studies, the correlation between end points was particularly strong (*R*^2^=0.93).

Several additional subset analyses were performed in an effort to determine if patient characteristics or study type affected results. Studies in which progression was delayed by at least 6 months in the standard group might represent patients with better prognosis than in the overall group of studies. However, analysis of this group of studies and analysis of studies with at least 100 patients per group were very similar to the overall analysis.

We also reran the overall analysis with indicators for blinding and availability of ITT results. Neither had a significant effect on the regression (*P*=0.77 and 0.36 respectively), nor did the parameter estimate for the slope of the progression end point change appreciably. The fact that a study reported HRs is another potential indicator for study quality. HRs from proportional hazards modeling properly account for censored data and may have incorporated additional covariate effects; the reported values provide the best effect estimates from each study. For the 10 studies that reported hazard ratios, the reported HR_TTP_ corresponded more closely with HR_OS_ (slope=0.52) than in the overall analysis using derived HRs and more of the variability was accounted for by the model (*R*^2^=0.52).

Additional exploratory analyses using different configurations of the outcome variable (difference in survival months, logarithm of the hazard ratio, analysis on survival months from separate treatment groups) provided results and inferences consistent with the models shown here.

## Discussion

These results demonstrate a clear positive association between effects on progression and survival in MBC patients, which is statistically significant. The analyses suggest that a treatment that prolongs time-to-progression will likely also have a longer overall survival time than the alternate treatment, although the relative effect will be substantially smaller. The analysis suggests that additional survival time seems to occur prior to progression and be a direct result of delaying this event; however, we did not have adequate data to look specifically at survival after progression.

The plot of effects on progression and survival illustrates the wide heterogeneity of results across studies conducted in metastatic breast cancer patients. In our analyses, we saw the highest level of predictability when analysing only the small subset of studies, which were conducted in HER2+ patients. Whether this finding is because of the similar design, homogeneous patients or treatment comparisons in these four studies remains in question. We note that the R-squared term (coefficient of determination) represents the amount of variance explained by the regression model and provides a measure of how reliably progression effects can predict survival effects. In other words, it measures the reproducibility of the relationship and is dependent both on the validity of the model and the sample size of the individual studies. On the other hand, the slope parameter (beta estimate) from the model quantifies the size of the effect, allowing one to ascertain how changes in progression effects will translate into changes in survival effects. In all the analyses presented here, we found a slope parameter that is statistically greater than zero (lower bound of CI is greater than zero). The relative survival effect is expected to be about one-third of the relative effect on progression.

Conducting a meta-analysis of time-to-event data from published reports is difficult as appropriate summary statistics are not always available in the publications. In practise, meta-analysis is constrained by data availability, often suffers from lack of power, and may not adequately capture the effects of patient characteristics. For this analysis, we made decisions regarding outcome selection, weighting and model building that allowed us to incorporate the most studies. Results should be viewed as hypothesis-generating, rather than confirmatory. Specific limitations related to reporting include the following:

- Inclusion criteria and individual patient characteristics of prognostic importance may have varied across studies. Thus, homogeneous groups or subgroups of patients cannot be readily identified.
- Studies had somewhat inconsistent definitions for progression end points. Methods of assessing progression and specifics about how progression itself was defined were not examined.
- Consistent summary statistics (for example, hazard ratios and variance) were not available for all trials making more detailed analyses impossible.
- Few studies clearly identified treatment options such as crossover after progression.

Although it seems almost obvious that progression-free survival should be correlated with overall survival, the link between delayed progression of MBC from anticancer drugs and survival benefit is not well established. The goal of this investigation was to explore the extent to which progression end points can provide a reliable surrogate for OS in trials conducted in women with metastatic breast cancer. Although reducing death from breast cancer must be the primary goal of clinical trials, use of OS as a primary end point has several problems. In particular, (1) there is no control of treatment options after progression has taken place, (2) survival results are delayed as progression precedes death by several years on average even in metastatic breast cancer, and (3) deaths from causes other than breast cancer may occur. All of these factors can dilute real treatment effects on breast cancer survival in these women. These diluting factors make it hard to confirm the link between effects on progression and effects on survival.

Results presented here are generally consistent with findings in other tumour types (Table 1), although the different analysis approaches and inclusion criteria make it difficult to compare results across the literature. In breast and other tumour types, the highest predictive power was achieved when meta-analyses were performed using individual patient data on a small group of trials (Sargent *et al*, 2005; Buyse *et al*, 2007). Hackshaw *et al* (2005) noted an association between treatment effects on progression and survival when comparing anthracycline-based regimens in advanced breast cancer and suggested use of time-to-progression as a surrogate marker for survival. Interestingly, they found a stronger relationship between the end points in studies performed prior to 1990 when second-line therapies for metastatic cancer were not commonly used. More recently, a meta-analysis of 11 studies in metastatic breast cancer comparing anthracyclines to taxanes examined the association of several end points with survival (Burzykowski *et al*, 2008). The researchers found tumour response to be more highly associated with overall survival than was progression-free survival. However, that meta-analysis appears to be unweighted. Our weighted analysis of anthracycline studies produced an *R*^2^ value almost double that reported by Burzykowski *et al* (2008). Using sample sizes as weights, we show a moderately strong linear correlation between progression-free survival and survival, in a large group of studies covering many treatment types. We find it intriguing that the strength of the relationship between end points may vary by tumour and/or treatment types and by line of therapy.

The consistency of findings in this comprehensive meta-analysis shows that we can expect overall survival with metastatic breast cancer to be extended when tumour progression is delayed, although the effect size will typically be smaller. The attenuated effect is due at least partly to the diluting factors indicated above and suggests that overall survival itself is not a perfect end point for assessing the value of a treatment. We do not believe that the current findings are sufficient for accepting time-to-progression as a fully validated surrogate marker for overall survival in MBC, as the variance in the relationship is not fully explained. Factors that could improve the prediction remain under investigation. Nonetheless, the evidence suggests that progression is a useful early end point that should feature prominently in assessments of treatment efficacy for MBC. Further work is needed to better understand what determinants are associated with the link between disease progression and breast cancer survival and whether some tumour and/or treatment subgroups experience a more substantial survival benefit from delayed progression.

## Figures and Tables

**Table 1:** Published meta-analyses in multiple tumour types

**Tumour type**	**Studies included**	**Reference**	**Relationship analysed**	**Number of studies**	**Slope coefficient**	***R* ^2^**
Breast: metastatic	All randomised studies reporting progression and survival times by treatment	Current analysis	Ratio of median progression and ratio of median survival times (centred at zero)	67	0.32	0.30
Breast: metastatic	Studies of anthracyclines and taxanes with individual patient data	Burzykowski *et al* (2008)	Log hazard ratio for progression and log hazard ratio for survival	11	Not provided	0.23
Breast: metastatic	Studies of epirubicin regimens with individual patient data	Bruzzi *et al* (2005)	Log hazard ratio for response and log odds ratio for survival	10	Not provided	0.10
Breast: advanced	Studies of first-line treatments using FAC or FEC	Hackshaw *et al* (2005)	Log hazard ratio for time-to-progression and log hazard ratio for survival	17 before 1990	0.58 before 1990	0.67 before 1990
				9 after 1990	0.40 after 1990	0.41 after 1990
Breast: adjuvant	Studies reporting 2- to 3-year disease-free survival and 5-year overall survival (*N*⩾100)	Ng *et al* (2008)	Difference in proportion with disease-free survival at 2 years and difference in proportion surviving >5 years	126	Not provided	0.38
Colorectal: advanced	Studies of FU+leucovorin with individual patient data	Buyse *et al* (2007)	Log hazard ratio for progression- free survival and log hazard ratio for survival	10	0.81	0.98
Colorectal: metastatic	Studies with ‘mature data’ (*N*⩾100)	Tang *et al* (2007)	Hazard ratio for progression and hazard ratio for survival	39	Not provided	0.55 (PFS) 0.27 (TTP)
Colorectal: metastatic	Studies of first-line treatments	Johnson *et al* (2006)	Difference in months-to-progression and difference in survival months	146	0.096	0.33
Colorectal: metastatic	Studies of first-line treatments (*N*⩾100 per arm)	Louvet *et al* (2001)	Median months progression-free survival and median months survival by treatment group	29	0.68	0.23
Colon: adjuvant	Studies selected based on ‘relevance, maturity and data availability’ (individual patient data)	Sargent *et al* (2005)	Hazard ratio for disease-free survival and hazard ratio for survival	18	0.89	0.90
Lung (non-small-cell)	Studies reporting hazard ratios since 1977	Johnson *et al* (2004)	Log hazard ratio for time-to-progression and log hazard ratio for survival	48	Not provided	0.42
Lung: metastatic	Studies of first-line treatments	Johnson *et al* (2006)	Difference in months-to-progression and difference in survival months	191	0.62	0.19

*R*^2^=coefficient of determination for multivariate analysis or derived as squared correlation coefficient.

**Table 2:** Treatment types in included studies of monotherapies (*n*=28)

	**Experimental monotherapy**					
**Comparator**	**Anthracycline**	**Chemotherapy**	**Hormonal agent**	**Taxane**	**Biologic**	**Other**
Anthracycline	4			2		
Chemotherapy		1		4		
Hormonal agent			10			
Taxane				3		
Biologic					1	
Placebo or usual care			1	1		1

**Table 3:** Treatment types in included studies of combination therapies (*n*=39)

	**Experimental combination regimens**							
**Comparator**	**Anthracycline+ taxane**	**Anthracycline+ chemotherapy**	**Anthracycline+ hormonal agent or biologic**	**Multiple anthracyclines**	**Multiple chemotherapies**	**Chemotherapies+ Other**	**Multiple hormonal agents**	**Biologic+ Chemotherapy or taxane**
Anthracycline+taxane	4							
Anthracycline+ chemotherapy	6	5	1		2			
Anthracycline+ other						1		
Anthracycline		3	1	2				
Chemotherapy		4			3	2		1
Hormonal agent							1	
Taxane	1							1
Biologic+taxane								1

**Table 4:** Summary of regression analyses^a^

	**Number of studies**	**Slope parameter estimate (95% CI)**	***R*^2^ (adjusted)**
Primary model – all studies	67	0.32 (0.20, 0.43)	0.30
Studies of hormonal treatments	12	0.58 (0.001, 1.17)	0.24
Studies of anthracyclines	36	0.32 (0.19, 0.43)	0.43
Studies of first-line treatments for MBC	46	0.29 (0.15, 0.42)	0.28
Studies of non first-line treatments	21	0.38 (0.14, 0.62)	0.32
Studies with only HER2+patients	4	0.50 (0.28, 0.71)	0.93
Studies which reported HRs	10	0.52 (0.18, 0.86)	0.52
Studies where TTP in standard group ⩾6 months	37	0.32 (0.18, 0.47)	0.35
Studies with N per group ⩾100	36	0.31 (0.16, 0.47)	0.31

CI=confidence interval.

a Models without intercept and weighted by sample size.

**Table 5:** Predicted HR_OS_ based on observed HR_TTP_

**Observed HR_TTP_**	Predicted^*^ HR_OS_
0.5	0.82 (0.76, 0.88)
0.6	0.85 (0.80, 0.90)
0.7	0.88 (0.84, 0.92)
0.8	0.91 (0.87, 0.95)

a Using model Effect OS=−0.03+(0.30 ^*^Effect TTP).

## References

[bib1] Bruzzi P, Del Mastro L, Sormani MP, Bastholt L, Danova M, Focan C, Fountzilas G, Paul J, Rosso R, Venturini M (2005) Objective response to chemotherapy as a potential surrogate end point of survival in metastatic breast cancer patients. J Clin Oncol 23: 5117–5125 15955906 10.1200/JCO.2005.02.106

[bib2] Burzykowski T, Buyse M, Piccart-Gebhart MJ, Sledge G, Carmichael J, Luck HJ, Mackey JR, Nabholtz JM, Paridaens R, Biganzoli L, Jassem J, Bontenbal M, Bonneterre J, Chan S, Basaran GA, Therasse P (2008) Evaluation of tumor response, disease control, progression-free survival, and time to progression as potential surrogate end points in metastatic breast cancer. J Clin Oncol 26: 1987–1992 18421050 10.1200/JCO.2007.10.8407

[bib3] Buyse M, Burzykowski T, Carroll K, Michiels S, Sargent DJ, Miller LL, Elfring GL, Pignon JP, Piedbois P (2007) Progression-free survival is a surrogate for survival in advanced colorectal cancer. J Clin Oncol 25: 5218–5224 18024867 10.1200/JCO.2007.11.8836

[bib4] Eiermann W (2001) Trastuzumab combined with chemotherapy for the treatment of HER2-positive metastatic breast cancer: pivotal trial data. Ann Oncol 12(Suppl 1): S57–S62 11521723

[bib5] Hackshaw A, Knight A, Barrett-Lee P, Leonard R (2005) Surrogate markers and survival in women receiving first-line combination anthracycline chemotherapy for advanced breast cancer. Br J Cancer 93: 1215–1221 16278665 10.1038/sj.bjc.6602858 PMC2361525

[bib6] Johnson K, Hill S, Stokes B, Berlin J, Freemantle N, Anthony D (2004) Developing Methodology for Validation and Comparison of Surrogate Markers in Randomized Clinical Trials (abstract). In 12th Cochrane Colloquium: Ottawa, Ontario, Canada

[bib7] Johnson KR, Ringland C, Stokes BJ, Anthony DM, Freemantle N, Irs A, Hill SR, Ward RL (2006) Response rate or time to progression as predictors of survival in trials of metastatic colorectal cancer or non-small-cell lung cancer: a meta-analysis. Lancet Oncol 7: 741–746 16945769 10.1016/S1470-2045(06)70800-2

[bib8] Kröger N, Frick M, Gluz O, Mohrmann S, Metzner B, Jackisch C, Ko Y, Lindemann HW, Meier CR, Lohrmann HP, Ruffert U, Hanel M, Bodenstein H, Neubauer A, Ehninger G, Wolf HH, Kolbe K, Burock K, Zander AR, Nitz U (2006) Randomized trial of single compared with tandem high-dose chemotherapy followed by autologous stem-cell transplantation in patients with chemotherapy-sensitive metastatic breast cancer. J Clin Oncol 24: 3919–3926 16921043 10.1200/JCO.2005.04.0352

[bib9] Lotz JP, Cure H, Janvier M, Asselain B, Morvan F, Legros M, Audhuy B, Biron P, Guillemot M, Goubet J, Laadem A, Cailliot C, Maignan CL, Delozier T, Glaisner S, Maraninchi D, Roche H, Gisselbrecht C (2005) High-dose chemotherapy with haematopoietic stem cell transplantation for metastatic breast cancer patients: final results of the French multicentric randomised CMA/PEGASE 04 protocol. Eur J Cancer 41: 71–80 15617992 10.1016/j.ejca.2004.09.006

[bib10] Louvet C, de Gramont A, Tournigand C, Artru P, Maindrault-Goebel F, Krulik M (2001) Correlation between progression free survival and response rate in patients with metastatic colorectal carcinoma. Cancer 91: 2033–2038 11391582

[bib11] Ng R, Pond GR, Tang PA, MacIntosh PW, Siu LL, Chen EX (2008) Correlation of changes between 2-year disease-free survival and 5-year overall survival in adjuvant breast cancer trials from 1966 to 2006. Ann Oncol 19: 481–486 18029973 10.1093/annonc/mdm486

[bib12] Sargent DJ, Wieand HS, Haller DG, Gray R, Benedetti JK, Buyse M, Labianca R, Seitz JF, O'Callaghan CJ, Francini G, Grothey A, O'Connell M, Catalano PJ, Blanke CD, Kerr D, Green E, Wolmark N, Andre T, Goldberg RM, De Gramont A (2005) Disease-free survival *vs* overall survival as a primary end point for adjuvant colon cancer studies: individual patient data from 20 898 patients on 18 randomized trials. J Clin Oncol 23: 8664–8670 16260700 10.1200/JCO.2005.01.6071

[bib13] Schmid P, Schippinger W, Nitsch T, Huebner G, Heilmann V, Schultze W, Hausmaninger H, Wischnewsky M, Possinger K (2005) Up-front tandem high-dose chemotherapy compared with standard chemotherapy with doxorubicin and paclitaxel in metastatic breast cancer: results of a randomized trial. J Clin Oncol 23: 432–440 15659490 10.1200/JCO.2005.06.072

[bib14] Slamon DJ, Leyland-Jones B, Shak S, Fuchs H, Paton V, Bajamonde A, Fleming T, Eiermann W, Wolter J, Pegram M, Baselga J, Norton L (2001) Use of chemotherapy plus a monoclonal antibody against HER2 for metastatic breast cancer that overexpresses HER2. N Engl J Med 344: 783–792 11248153 10.1056/NEJM200103153441101

[bib15] Tang PA, Bentzen SM, Chen EX, Siu LL (2007) Surrogate end points for median overall survival in metastatic colorectal cancer: literature-based analysis from 39 randomized controlled trials of first-line chemotherapy. J Clin Oncol 25: 4562–4568 17876010 10.1200/JCO.2006.08.1935

